# Chronic Kidney Disease among COVID-19 Patients Admitted in the Department of Medicine of a Tertiary Care Centre: A Descriptive Cross-sectional Study

**DOI:** 10.31729/jnma.8167

**Published:** 2023-05-31

**Authors:** Milan Khadka, Lochan Karki, Rama Tamrakar, Milan Purna Oli, Santosh Joti, Suman Khatri, Siddhant Adhikari, Shrinkhala Maharjan, Poonam K C

**Affiliations:** 1 Department of Medicine, National Academy of Medical Sciences, Mahaboudha, Kathmandu, Nepal; 2 Lubhoo Primary Health Care Centre, Mahalaxmi, Lalitpur, Nepal; 3 Godawari Midcity Hospital, Satdobato, Lalitpur, Nepal; 4 Silverline Hospital, Balaju, Kathmandu, Nepal; 5 Lumbini Provincial Hospital, Butwal, Rupandehi, Nepal; 6 Clinic Health Care Centre, Balaju, Kathmandu, Nepal; 7 Ganeshman Singh Memorial Hospital and Research Center, Mahalaxmisthan, Lalitpur, Nepal

**Keywords:** *chronic kidney disease*, *COVID-19*, *prevalence*, *tertiary care centre*

## Abstract

**Introduction:**

Comorbidities are frequently seen in admitted COVID-19 patients most common being hypertension, diabetes, cardiovascular diseases and chronic kidney disease. Chronic kidney disease is a slowly progressive chronic illness due to the gradual loss of kidney function or structure. The available data regarding the prevalence of chronic kidney disease and COVID-19 comorbidities is still limited. The aim of this study was to find out the prevalence of chronic kidney disease among COVID-19 patients admitted to the Department of Medicine of a tertiary care centre.

**Methods:**

A descriptive cross-sectional study was done in the Department of Medicine of a tertiary care centre. Data of medical records between 1 August 2020 to 1 December 2022 were reviewed retrospectively. The data was collected from 20 January 2023 to 20 March 2023. Ethical approval was obtained from the Institutional Review Committee (Reference number: 646/2079/80). Data on chronic kidney disease patients among COVID-19 patients were collected from the hospital records. Convenience sampling method was used. Point estimate and 95% Confidence Interval were calculated.

**Results:**

Among 584 COVID-19 patients admitted, the prevalence of chronic kidney disease was 43 (7.36%) (5.24-9.48, 95% Confidence Interval). A total of 30 (69.77%) were male and 13 (30.23%) were female with a mean age of 55±16.22 years.

**Conclusions:**

The prevalence of chronic kidney disease among COVID-19 patients admitted in the department of Medicine of a tertiary care centre was found to be slightly higher than other studies done in similar settings.

## INTRODUCTION

The Wuhan city of China evidenced unknown aetiology pneumonia cases at the end of December 2019. On 7 January 2020, the causative agent was identified as (Severe acute respiratory syndrome coronavirus 2) SARS-CoV-2, and the disease as Coronavirus disease (COVID-19).^1^ The 2019 novel coronavirus presents a wide spectrum of clinical disease presentations, from asymptomatic infection to respiratory failure with high mortality.^2,3^

Comorbidities are frequently seen in admitted COVID-19 patients most common being hypertension, diabetes, cardiovascular diseases and chronic kidney disease (CKD).^4^ Chronic kidney disease is a slowly progressive chronic illness due to the gradual loss of kidney function or structure.^5^ Although evidence from recent studies suggests that individuals with preexisting comorbidities are at a greater risk of mortality due to COVID-19, the available data regarding the association between COVID-19 and underlying comorbidities is still limited.^6,7^

The aim of this study was to find out the prevalence of CKD among COVID-19 patients admitted to the Department of Medicine of a tertiary care centre.

## METHODS

A descriptive cross-sectional study was conducted among COVID-19 patients admitted to the Department of Medicine, National Academy of Medical Sciences, Mahaboudha, Kathmandu, Nepal. Data of medical records between 1 August 2020 to 1 December 2022 were reviewed retrospectively. The data was collected from 20 January 2023 to 20 March 2023. Ethical approval was obtained from the Institutional Review Committee of the same institute (Reference number: 646/2079/80). All the COVID-19 patients diagnosed via nasopharyngeal and/or oropharyngeal swabs with polymerase chain reaction,^8^ admitted to the Department of Medicine of the National Academy of Medical Sciences from age 15 years and above were included in the study. Those with incomplete data and leave against medical advice were excluded from the study. Convenience sampling method was used. The sample size was calculated using the following formula:


$$\begin{array}{ll} \text{n} & ={\text{Z}}^{2}\times \frac{\text{p}\times \text{q}}{{\text{e}}^{\text{2}}}\\ & =1.96^{2}\times \frac{0.065\times 0.935}{0.02^{2}}\\ & =584 \end{array}$$

Where,

n = minimum required sample size
Z = 1.96 at 95% Confidence Interval (CI)
p = prevalence of CKD among COVID-19 patients, 6.5%^4^
q = 1-p
e = margin of error, 2%

The calculated sample size was 584. A convenience sample method was used. CKD is defined as progressive loss of kidney function for more than or equal to three months with a glomerular filtration rate (GFR) lower than 60 ml/min/1.73 m^2^, or GFR greater than 60 ml/min/1.73 m^2^, with evidence of renal structure injury.^5^

Data was entered in Microsoft Excel 2016 and analysis was done using IBM Statistics SPSS 26.0. Point estimate and 95% CI were calculated.

## RESULTS

Among 584 COVID-19 patients, the prevalence of CKD was 43 (7.36%) (5.24-9.48, 95% CI).

A total of 30 (69.77%) were male and 13 (30.23%) were female and the mean age among CKD patients was 55±16.22 years (Table 1).

**Table 1:** Demographic distribution (n = 43).

Sex	n (%)
Male	30 (69.77)
Female	13 (30.23)
**Age Group (years)**	
15-30	3 (6.90)
31-45	9 (21)
46-60	12 (28)
61-75	17 (39.50)
>75	2 (4.60)

A total of total 12 (27.90%) patients with CKD did not have other comorbidities while 31 (72.10%) patients with CKD had at least one other comorbidity. Among them, hypertension and diabetes mellitus were the most common, which were present in 26 (60.46%) patients and 16 (37.20%) respectively (Table 2).

**Table 2:** Other Comorbidities among COVID-19 patients with CKD (n= 43).

Comorbidity	n (%)
Hypertension	26 (60.46)
Diabetes mellitus	16 (37.2)
Cardiac disease	3 (7)
Others	8 (18.60)

**Figure 1:**
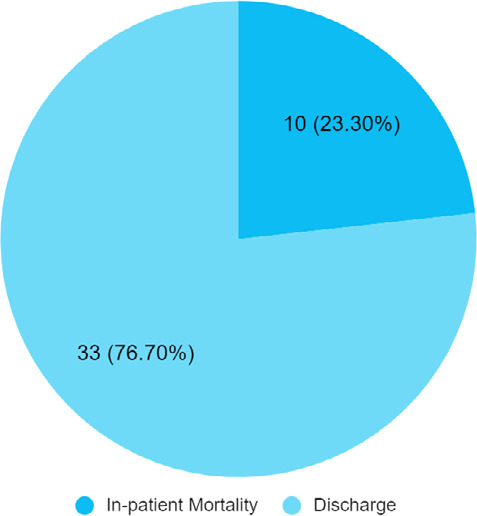
Outcome of COVID-19 patients with CKD (n = 43).

Among 43 COVID-19 patients with CKD, in-hospital mortality was 10 (23.30%) (Figure 1).

## DISCUSSION

Among 584 COVID-19 patients from 15 years and above hospitalised in the department of medicine of a tertiary care centre, the prevalence of CKD was found to be 7.36% which is slightly higher in comparison to other studies, which ranges from 0.8 - 6.5 %.^4,6,9-11^ A study done among 445 patients in Lahore, Pakistan concluded that CKD was seen in 6.5% of COVID-19 patients.^4^ A study conducted in Hyderabad, India showed that 0.8 % of COVID-19 patients had CKD as a comorbidity.^6^ Another similar study done in Karachi, Pakistan showed that CKD as a comorbidity was seen in 2.83% of COVID-19 patients.^9^ In A nationwide study done among 1590 hospital-admitted COVID-19 patients in China showed that 2% of patients have CKD as a comorbidity.^10^ In other observation studies sought from MEDLINE, Embase and the Cochrane Library, the prevalence of CKD was 5.2 %.^11^

In our study, among COVID-19 patients with CKD, the mean age group was found to be 55±16.22 years and among them, 69.77% were male and 30.23% were female. The mean age group in the multicenter observational study conducted in Japan was 63±16 years which was slightly higher than our study, among which 70.8% were male and 29.2% were female which was similar to our study, whereas, hypertension and diabetes were seen among 47.4% and 26.2% respectively.^12^

In a retrospective study conducted in three affiliated hospitals in New York City among COVID-19 patients with CKD, 69% were male and 31% were female. In the same study, among COVID-19 patients with CKD having different comorbidities were found to be hypertension (83%), and DM (37%). In our study, hypertension was 60.47%, and DM was 37.21%.^13^

In our study, in-hospital mortality was seen in 23.3% whereas 76.7% of patients were discharged from the hospital. In a similar study done in Lahore, Pakistan, mortality was seen in 24.1% of patients whereas recovery was seen in 75.9% of patients which was similar to our study findings.^4^

The mean duration of hospital stay in this study was 8.83±6 days and the number of days of hospital stay varied from 1 to 30. A similar study done in Iran showed a hospital stay of 11.65±6.67 days and the number of days varied from 2 to 33 days.^14^ The findings in our study are similar to the study done in Iran.

The limitations of our study was that it was conducted in an exclusive single hospital setting which is a tertiary care government hospital located in the capital city. Thus, the findings may not be generalisable. Therefore, the study's limitations should be taken into consideration before any application of the findings. Since we have done a descriptive cross-sectional study, we could not determine CKD as an independent risk factor for increased morbidity and mortality among COVID-19 patients. Further analytical studies are required to find out the correlation.

## CONCLUSIONS

The prevalence of CKD among COVID-19 patients admitted to the department of Medicine of a tertiary care centre is slightly higher than other studies done in similar settings.
